# Electronic Structure and Lattice Engineering of Cobalt Doping FeS_2_@C for Superior Electrosorption of Ytterbium Ions

**DOI:** 10.3390/ma18214994

**Published:** 2025-10-31

**Authors:** Kaicheng Bi, Tiancai Cheng, Zhangjie Shi, Wenyan Huang, Fuli Deng, Yi Zhang

**Affiliations:** 1 State Key Laboratory of Green and Efficient Development of Phosphorus Resources, Key Laboratory of Green Chemical Process of Ministry of Education, Hubei Key Laboratory for Novel Reactor and Green Chemical Technology, Engineering Research Center of Phosphorus Resources Development and Utilization of Ministry of Education, School of Chemical Engineering and Pharmacy, Wuhan Institute of Technology, Wuhan 430073, China; fenhuie445@163.com (K.B.); ctc1102637537@163.com (T.C.); szj1239695449@163.com (Z.S.); huangwenyan0616@163.com (W.H.); 2 Hubei Three Gorges Laboratory, Yichang 443007, China

**Keywords:** capacitive deionization, electrosorption, rare earth, selectivity, pyrite

## Abstract

Facing the increasingly scarce supply of rare-earth resources, a cobalt-doped metal–organic framework-derived carbon–metallic sulfide composite (Co-FeS_2_@C) was successfully synthesized via the hydrothermal method and the following carbonization/sulfidation treatments and used for the efficient electrosorption of rare earths from aqueous solution. Comparative characterizations revealed that Co doping effectively expanded the interlayer spacing of FeS_2_, introduced crystalline defects, and optimized the electronic structure, thereby synergistically enhancing active site exposure and electron transfer kinetics. In addition, the electrochemical analysis demonstrated a significant increase in the surface-controlled capacitive contribution from 57.1% to 83.3%, indicating the markedly improved electric double-layer effects and mass transport efficiency. Under the optimal conditions, the Co-FeS_2_@C electrode achieved a high Yb^3+^ adsorption capacity of 129.2 mg g^−1^ along with an exceptional cycling stability (92.63% retention after 20 cycles), substantially outperforming the undoped counterpart FeS_2_ (88.4 mg g^−1^ and 74.61%). Furthermore, the mechanistic investigations confirmed that the electrosorption process follows a monolayer physico-chemical synergistic mechanism, primarily driven by the pseudo-capacitive effect arising from the redox reaction of FeS_2_ and the enhanced charge-transfer driving force resulting from the higher electronegativity of cobalt. This work provides an innovative electronic structure modulation strategy for developing the high-performance capacitive deionization electrodes for rare earth recovery via the electrosorption process.

## 1. Introduction

As critical strategic non-renewable resources, the development and utilization of rare earths has always been seriously regarded in various countries and research areas [1,2]. In order to obtain high-purity rare earths, the purification and separation of rare earth elements can be achieved via traditional extraction technology [3,4]. However, processing wastewater containing incompletely separated rare earths and other harmful substances is inevitably generated during the extracting process. Discharging the wastewater will pollute the soil and water resources and thus jeopardize the health of human beings, and also lead to the substantial loss of rare earth resources [5]. Currently, a variety of methods have been devoted to recovering the rare earth elements from the processing wastewater, such as co-precipitation, adsorption, and membrane separation [6,7,8,9]. Among them, adsorption is considered the most feasible method in terms of its operation and economic practicability [10]. However, various obvious drawbacks, such as poor selectivity, limited adsorption capacity, and discontinuous operation, also limit the application of adsorption methods in practical water treatment. Nowadays, capacitive deionization (CDI), as an emerging technology for ion extraction from aqueous solution, provides a novel strategy for the recovery of rare earth ions from aqueous solution due to its low energy consumption, high-efficient desorption, simple operation, and good cyclic stability [11,12].

CDI technology works by applying a low voltage on both sides of the electrode materials to create the effect of the double electric layer (EDL) and thus the adsorption of ions from aqueous solution [13]. The design of the electrode material is crucial in CDI technology. Carbon materials are an important component of electrode materials, including activated carbon, carbon nanotubes, carbon aerogel, and graphene, due to their large specific surface area and excellent electrical conductivity [14,15,16]. However, carbon materials also exhibit low selectivity and poor electrosorption ability and regeneration performance due to their limited active sites for ion storages, which limits their development and practical application [17,18]. In recent years, metal–organic frameworks (MOFs) have gradually been applied as the self-sacrificial templates, and widely used in electrocatalytic and energy storage systems due to their tunable surface area, rich carbon content, and various loaded metals [19,20]. To further enhance the capacitive electrosorption capacity of MOF-derived materials, the transition metal oxides are utilized as pseudocapacitive electrode materials to obtain more desirable electrosorption capabilities through redox reactions or ion intercalation effects [21]. However, the inherent poor electronic conductivity of the metal oxides hinders electron transfer, leading to the deactivation of the electrosorption sites, resulting in slow electrosorption rates and poor electrosorption cycle stability [22,23]. In addition, the S atom, with greater electronegativity and atomic radii than oxides, endows the transition metal sulfides with higher electronic conductivity and stronger electrosorption capacity [24]. In particular, the first-row transition metal sulfides exhibit impressive electrochemical activities in different electrochemical fields. More importantly, S doping in carbon materials can also improve the selectivity for metal ions by forming S-cluster covalent bonds on Lewis bases to bind metal ions, thus demonstrating the greater advantage of transition metal sulfides when compared to the corresponding oxides [25,26].

Pyrite (FeS_2_) is one of the most ideal materials for capacitive deionization electrodes due to its unparalleled theoretical capacity (896 mAh g^−1^) [27]. However, the electrochemical performance of FeS_2_ is still limited by a lack of rate capability and stability due to slow reaction kinetics [28]. Accordingly, many efforts have been made to improve its electrochemical performance by introducing different heteroatoms (i.e., N, P, and Se) into FeS_2_, which obviously alters the charge distributions and lattice distances, thereby promoting charge transfer rates and generating more electrochemically active sites [29,30,31,32]. Therefore, the superior conductivity of electrode materials facilitates the electrosorption kinetics and optimize the intrinsic activity of S, resulting in excellent rate capability, specific capacity, and stability [33,34,35]. Currently, the co-doping of carbon and transition metal sulfides through the introduction of transition metal cations has been demonstrated to effectively enhance the conductivity of FeS_2_, optimize its electron distribution, and improve ion adsorption energy [36]. Among various candidates, cobalt is regarded as one of the most ideal dopants for FeS_2_ due to its ability to effectively suppress FeS_2_ oxidation, optimize the d-p band center [37], and enhance electrochemical activity [38]. Nevertheless, the application of cobalt as a dopant to enhance CDI technology has rarely been reported.

Therefore, this study employed iron-based MIL-101(Fe) (a metal–organic framework with a three-dimensional porous structure [39]) and its cobalt-doped derivative MIL-101(Co, Fe) as precursors to successfully prepare corresponding sulfides (namely FeS_2_@C and Co-FeS_2_@C) through a two-step process involving carbonization and subsequent sulphuration. Furthermore, the effects of Co-doping FeS_2_@C on its structural characteristics and their electrochemical analysis were comprehensively discussed. Subsequently, the optimal CDI conditions were explored, including the initial voltage, the concentration and pH of rare earth solution, the operating time, and selectivity. In addition, the CDI mechanism of Co-FeS_2_@C was also elucidated via XPS analysis, which provides a novel strategy for optimizing the CDI materials.

## 2. Experimental Section

### 2.1. Materials

Iron(III) chloride hexahydrate (FeCl_3_⋅6H_2_O), cobalt(II) chloride hexahydrate (CoCl_2_·6H_2_O), terephthalic acid, N,N-Dimethylformamide (DMF), ytterbium nitrate hexahydrate (Yb(NO_3_)_3_⋅6H_2_O), lanthanum nitrate hexahydrate (La(NO_3_)_3_⋅6H_2_O), neodymium nitrate hexahydrate (Nd(NO_3_)_3_⋅6H_2_O), samarium nitrate hexahydrate (Sm(NO_3_)_3_⋅6H_2_O), gadolinium nitrate hexahydrate (Gd(NO_3_)_3_⋅6H_2_O), dysprosium nitrate pentahydrate (Dy(NO3)_3_⋅5H_2_O), methanol, ethanol, ultrapure water, polyvinylidene fluoride, acetylene black, and 1-methyl-2-pyrrolidone were obtained from Sinopharm Co., Ltd., Beijing, China. All chemical reagents were of analytical grade and used without further purification.

### 2.2. Synthesis of MIL-101(Fe) and MIL-101(Co, Fe)

MIL-101(Co, Fe) was prepared according to the previous reports with some modifications [40]. A measure of 1.08 g of FeCl_3_⋅6H_2_O (4 mmol), different amounts of CoCl_2_·6H_2_O, and 0.332 g of terephthalic acid (2 mmol) were dispersed into 60 mL of DMF under magnetic stirring for 1 h to form a clear solution. The solution was then transferred to a PTFE-lined autoclave (100 mL) and kept at 110 °C for 24 h. After naturally cooling to the ambient temperature, the product was filtered and washed with DMF and ethanol. Finally, the solid powder was collected after being dried in a vacuum oven at 60 °C overnight. In addition, MIL-101(Fe) was synthesized via the same procedure without the addition of CoCl_2_⋅6H_2_O.

### 2.3. Synthesis of Co-FeS_2_@C and FeS_2_@C

MIL-101(Co, Fe) was calcined at 600 °C with a heating rate of 5 °C min^−1^ under Ar for 2 h to obtain Co-Fe_3_O_4_@C. Then, Co-Fe_3_O_4_@C and sulfur powder with the mass ratio of 1:6 was placed in two different crucibles in a tube furnace, respectively and Co-FeS_2_@C was successfully fabricated by sulfurization at 500 °C with a heating rate of 5 °C min^−1^ for 2 h under Ar. In addition, FeS_2_@C was also obtained by converting MIL-101(Fe) via successive carbonization and sulfurization.

### 2.4. Characterizations

The physicochemical properties of the studied electrode materials were systematically analyzed using various characterization techniques. Field emission scanning electron microscopy (FESEM, Gemini 300, Carl Zeiss AG, Oberkochen, Germany) and transmission electron microscopy (TEM, Tecnai G2F30, FEI Company, Hillsboro, OR, USA) revealed the surface morphology and internal crystal structure of the materials, respectively. Energy dispersive X-ray spectroscopy (EDS) provided results related to the elemental distributions. The crystalline phase composition of the materials was analyzed by X-ray diffraction (XRD, Bruker D2 PHASER, Bruker Corporation, Bremen, Germany) using Cu-Kα radiation over a 2θ range of 5–80° operated at 40 kV and 40 mA. Fourier transform infrared (FTIR) spectra were acquired using a Thermo Nicolet IS5 spectrometer (Thermo Fisher Scientific, Waltham, MA, USA) with a spectral resolution of 4 cm^−1^, employing the KBr pellet method with sample and KBr weights of approximately 1 mg and 300 mg, respectively. Electron paramagnetic resonance (EPR) analysis was performed on a Bruker A300-10/12 spectrometer (Bruker Corporation, Bremen, Germany) to characterize vacancy defects in the materials. The typical instrumental parameters were set as follows: microwave frequency 9.32 GHz, scan width 5000 G, time constant 40.96 ms, scan time 82 s, power 0.2 mW, and field-modulation amplitude 0.5 G. The Raman micro-spectroscopy (Raman microscope DXR, Thermo Fisher Scientific, Waltham, MA, USA) and X-ray photoelectron spectroscopy (XPS, Thermo Fisher Scientific, K-Alpha) were used to quantify the graphitized/amorphous carbon ratio and the chemical state of the surface elements in the carbon materials, respectively. The Raman micro-spectroscopy was performed using a 532 nm laser source. All spectra were collected at room temperature with a 50× objective lens. To prevent potential sample damage, the laser power incident on the sample surface was controlled at approximately 2 mW. Spectral acquisition involved 10 accumulation cycles, each with an integration time of 5 s. Changes in the concentration of metal ions in mixing rare earth solution were monitored by inductively coupled plasma emission spectroscopy (ICP-AES, Agilent 700, Agilent Technologies Inc., Santa Clara, CA, USA). The specific surface area and pore structure properties of the studied material were determined by the Brunauer–Emmett–Teller (BET) and Barrett–Joyner–Halenda (BJH) methods, respectively (Micromeritics, ASAP 2460, Micromeritics Instrument (Shanghai) Co., Ltd., Shanghai, China).

### 2.5. Electrochemical Measurements

Electrochemical performance was evaluated in a 1 mol L^−1^ Yb(NO_3_)_3_ electrolyte using a conventional three-electrode configuration. A working electrode was fabricated by coating a nickel foam substrate with a homogeneous slurry composed of active material, acetylene black, and polyvinylidene fluoride (PVDF) in an 8:1:1 mass ratio. A graphite rod and a Ag/AgCl electrode served as the counter and reference electrodes, respectively. Cyclic voltammetry (CV) measurements were conducted within a voltage window of −0.9 to −0.2 V vs. Ag/AgCl at scan rates ranging from 5 to 100 mV s^−1^. Galvanostatic charge–discharge (GCD) profiles and electrochemical impedance spectroscopy (EIS) were also performed, the latter being carried out at a bias voltage of 0.24 V over a frequency range from 100 kHz to 0.01 Hz. The specific capacitance (C_s_) was determined by integrating the area under the CV curves [27]:

(1) $$Cs=\frac{\int IdV}{2mv∆V}$$

In the equation above, *C_s_* (F g^−1^) is the specific capacitance, *I* (A) represents the current, *m* (g) and ∆*V* (V) are the material loading and the voltage window, respectively, and *v* (mV s^−1^) refers to the scanning speed of the CV test.

### 2.6. CDI Experiments

The CDI experiment focuses on the removal performance of Yb^3+^ as well as other rare earth ions. The active material slurry that was prepared by mixing active material, acetylene black, and PVDF at a mass ratio of 8:1:1, was uniformly coated on a 4 cm × 4 cm titanium plate to make a working electrode after being dried. The titanium plates loaded with active material slurry and activated carbon were connected to the negative and positive electrodes of the DC power supply, respectively, and assembled into a CDI device. The electrosorption behaviors were investigated using a flow system with a flow rate of 50 mL min^−1^ under the applied voltages of 0~1.2 V, pH values of 3.5~6, and Yb^3+^ initial concentration of 50~1000 mg L^−1^. The changes in solution conductivity were monitored in real-time by a conductivity meter (DDSJ-308F, Shanghai Yidian Scientific Instrument Co., Ltd., Shanghai, China). The electrosorption capacities were quantitatively analyzed by ICP-AES to systematically evaluate the selective electrosorption behavior when using the mixing solution including La^3+^, Nd^3+^, Sm^3+^, Gd^3+^, Dy^3+^, and Yb^3+^.

## 3. Results and Discussion

### 3.1. Characterizations

**Figure 1:**
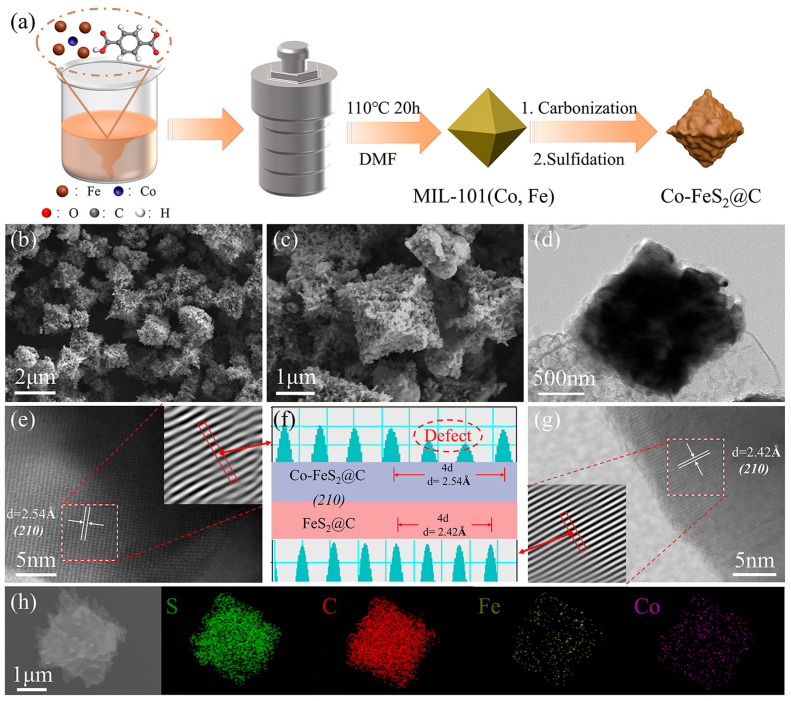
(**a**) The preparation route. (**b**–**d**) FESEM and TEM images of Co-FeS_2_@C. (**e**–**g**) HRTEM image and simulated lattice width of Co-FeS_2_@C and FeS_2_@C. (**h**) Elemental mapping images of Co-FeS_2_@C.

As shown in Figure 1a, MIL-101(Fe) and MIL-101(Co, Fe) were prepared by the hydrothermal method and continued to be converted into FeS_2_@C and Co-FeS_2_@C, respectively, via the carbonization and sulfidation treatments. Figure S1a shows the XRD patterns of MIL-101(Fe) and MIL-101(Co, Fe) samples. The positions of the main diffraction peaks are observed at 5.78°, 8.32°, 8.95°, and 16.36° for MIL-101(Fe), which are consistent with previous reports, which confirms the successful fabrication of MIL-101(Fe) [41]. The XRD characteristic peaks of MIL-101(Co, Fe) exhibit similarity to those of MIL-101(Fe), yet with notably enhanced intensity and reduced full width at half maximum for the primary diffraction peaks [39,42]. These observations collectively indicate that cobalt doping optimizes crystal growth and improves crystallinity. As shown in Figure S1b, the XRD patterns confirm that MIL-101(Fe) and MIL-101(Co, Fe) are converted into Fe_3_O_4_ and Co-doped Fe_3_O_4_, respectively, following annealing treatment. As observed in Figure S2, FESEM image shows the cuboctahedral morphology of MIL-101(Fe), with some holes on the surface, and MIL-101(Co, Fe) still maintains the cuboctahedral morphology with the shrinking surface after Co doping. This may be due to the fact that the introduction of Co can replace the original Fe-O bond and combine with Fe^3+^ to form a more stable Fe-Co metal bond, which reduces the lattice distortion of the material. In addition, Fe^3+^ tends to form octahedral coordination, but is prone to local distortion due to uneven distribution of ligands, whereas Co^2+^ has a more pronounced preference for octahedral coordination, which balances out the coordination asymmetry of Fe^3+^ and forms a more regular metal–ligand network [41,43].

**Figure 2:**
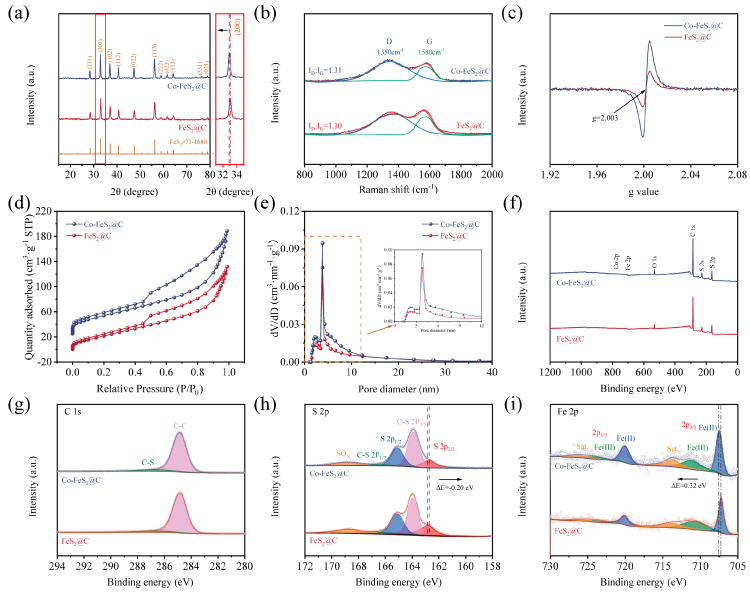
(**a**) XRD patterns. (**b**) Raman spectroscopy. (**c**) EPR spectra. (**d**,**e**) Nitrogen adsorption–desorption isotherms and corresponding pore size distribution. XPS spectra of (**f**) survey scan. High-resolution spectra of (**g**) C 1s, (**h**) S 2p, and (**i**) Fe 2p of FeS_2_@C and Co-FeS_2_@C.

As shown in Figure 1b,c, FESEM images demonstrate that Co-FeS_2_@C maintains the cuboctahedral morphology with the increased surface roughness. TEM images in Figure 1d and Figure S4c reveal that both Co-FeS_2_@C and FeS_2_@C exhibit the solid architectures. HRTEM images and simulated lattice width measurements (Figure 1e–g) show the distinct lattice spacings of 2.42 Å for FeS_2_@C, corresponding to the (210) plane of FeS_2_, and an increased spacing of 2.54 Å for the (210) plane in Co-FeS_2_@C, which are consistent with the observed negative-shift in characteristic peaks in the XRD pattern of Co-FeS_2_@C compared to pristine FeS_2_@C (Figure 2a). Subsequently, EDS analysis confirms the presence of Co, Fe, S, and C elements in Figure 1h, providing further evidence of successful cobalt doping [38].

As shown in Figure 2a, XRD characteristic peaks of FeS_2_ are observed at 28.5°, 33.0°, 37.1°, 40.7°, 47.4°, 56.3°, 59.0°, 61.7°, 64.3°, 76.6°, and 78.9°, which correspond to the peaks of (111), (200), (102), (112), (022), (113), (222), (023), (123), (331), and (024) crystal planes of FeS_2_, respectively (PDF#71-1680) [38]. Meanwhile, the negative shift in the characteristic peaks can be detected through the local zoomed-in image, which is due to the larger ionic radius of Co^2+^ (0.74 Å) compared to that of Fe^3+^ (0.64 Å), and the resultant larger spacing of crystal planes. In addition, no characteristic peaks corresponding to cobalt-containing compounds were detected in the Co-FeS_2_@C composite, collectively indicating successful incorporation of Co into the FeS_2_@C lattice via Co doping.

In order to investigate the carbon matrix structural changes in the material, Raman spectroscopy was used to verify the structural defective degree of carbon matrix in two materials. As shown in Figure 2b, the characteristic G and D bands at 1580 cm^−1^ and 1350 cm^−1^ generally represent the E_2_g optical phonon vibrational mode of carbon atomic bonds and the A_1_g symmetry breathing vibrational mode at graphite lattice boundaries or defect sites in carbon materials, respectively. The fitted area ratio (I_D_/I_G_) of D and G bands reveals the content of the material’s disordered carbon [27,44]. Generally, the larger I_D_/I_G_ indicates the higher the degree of disordered carbon in the material. It can be seen that I_D_/I_G_ increases from 1.10 to 1.31 after Co doping, suggesting that Co doping increases the amount of in disordered carbon in the material. Therefore, more structural defects and more active sites can be obtained in Co-FeS_2_@C after Co doping [45].

To further verify the presence of defects in the material structure, electron paramagnetic resonance (EPR) analysis was conducted on both materials. As shown in Figure 2c, Co-FeS2@C exhibits a stronger resonance signal at g = 2.003 compared to FeS_2_@C, indicating that Co doping introduces abundant defects [36,38]. This structural modification leads to rearrangement of atoms around the vacancies, resulting in locally destabilized regions and an increased number of active sites. These active sites facilitate electron migration within the electrode, thereby enhancing its electrochemical performance.

To further investigate the microstructures of MIL-101(Fe) and MIL-101(Co, Fe), it is essential to first elucidate the fundamental structural characteristics of MIL-101. This framework is a three-dimensional crystalline material constructed from metal trimeric clusters coordinated with terephthalic acid organic linkers. Within this structural context, FTIR spectroscopy was employed to characterize the functional groups of both materials. As shown in Figure S3, the FTIR spectra of these two MOFs are basically the same, showing the characteristic peaks at 544, 1388, 1600, and 1660 cm^−1^. These spectral signatures correspond to the stretching vibrations of metal-oxygen bonds (Fe-O/Co-O), the symmetric vibration of C-O bonds, the asymmetric vibration of C=O bonds, and the stretching vibrations of C=O bond, respectively [27,46,47]. The similar FTIR spectra indicate that Co is primarily incorporated into the MIL-101(Fe) framework via isomorphous substitution, wherein Co atoms replace a fraction of Fe^3+^ sites within the metal nodes. This substitution facilitates the formation of more stable Fe-Co metallic bonds, thereby optimizing the crystalline architecture and enhancing the structural stability of the MOFs.

To explore the specific surface area and pore distribution of studied materials in detail, BET measurements were conducted. The N_2_ adsorption–desorption isotherms of both materials exhibit distinct hysteresis loops that correspond to type IV isotherms, indicating their mesoporous nature (Figure 2d). The calculated specific surface areas of Co-FeS_2_@C and FeS_2_@C are determined to be 274 and 206 m^2^ g^−1^, respectively. As shown in Figure 2e, the comparative analysis of the pore size distribution profiles clearly demonstrates that cobalt doping effectively enhances the preservation of microporous and mesoporous structures during the annealing and sulfidation processes, thus optimizing the overall pore architecture of the material.

In addition, XPS was employed to investigate the electronic structure and surface chemical states of Co-FeS_2_@C and FeS_2_@C. As anticipated, Fe, Co, S, and C elements are detected in Co-FeS_2_@C (Figure 2f), consistent with the elemental mapping results. In the C 1s spectra in Figure 2g, the peaks at 286.8 eV and 284.8 eV are assigned to C-S and C-C bonds, respectively [27,48]. The high-resolution S 2p spectra in Figure 2h reveal two peaks at 164.8 eV (S 2p_1/2_) and 162.7 eV (S 2p_3/2_) for FeS_2_ and Co-FeS_2_@C. Meanwhile, another two peaks at 165.8 eV and 163.8 eV correspond to the C-S bond [49] and the peak at 168.9 eV is attributed to a SO_x_; species resulting from the oxidized material. Notably, upon Co doping, the relative intensity of the S 2p_1_/_2_ peak increases, while that of the S 2p_3_/_2_ peak decreases. Since the S 2p_1_/_2_ component is typically associated with low-coordinated sulfur related to sulfur vacancies, and the S 2p_3_/_2_ peak is attributed to metal–S bonds on the material surface [26,32], these spectral changes indicate that Co doping effectively enhances the concentration of sulfur vacancies in the material [29]. As illustrated in the Fe 2p spectra (Figure 2i), the characteristic peaks at 711.0 eV and 719.5 eV are identified as Fe^2+^, whereas the peaks of Fe^3+^ 2p_1/2_ and Fe^3+^ 2p_3/2_ are observed at 715.8 eV and 724.6 eV, respectively [50]. In addition, the corresponding satellite peaks for Fe 2p_1/2_ and Fe 2p_3/2_ are detected at 716.6 eV and 733.3 eV, respectively [51,52]. Notably, the Fe 2p peaks in Co-FeS_2_@C exhibited a positive shift toward higher binding energies (ΔE = 0.32 eV), which is attributed to the higher electronegativity of Co (1.88) relative to Fe (1.83) [53]. This shift indicates reduced electron density at Fe sites, enhancing their electron-accepting capability. Conversely, the S 2p_3/2_ peak displayed a negative shift, suggesting increased electron density at S sites. Quantitative analysis of Fe 2p peak areas reveals a higher Fe^3+^ content in Co-FeS_2_@C. Given that Fe^3+^ serves as an electrochemically active site, these results demonstrate that Co doping solidly optimizes the electronic configuration of FeS_2_, inducing charge redistribution among elements. Such modifications enhance the electrochemical performance of the material, which likely contributes to improved CDI capabilities [54,55]. As shown in Figure S5, a distinct Co 2p peak at 780.5 eV in Co-FeS_2_@C further confirms the successful Co doping [56,57].

### 3.2. Electrochemical Measurements of Electrode Materials

**Figure 3:**
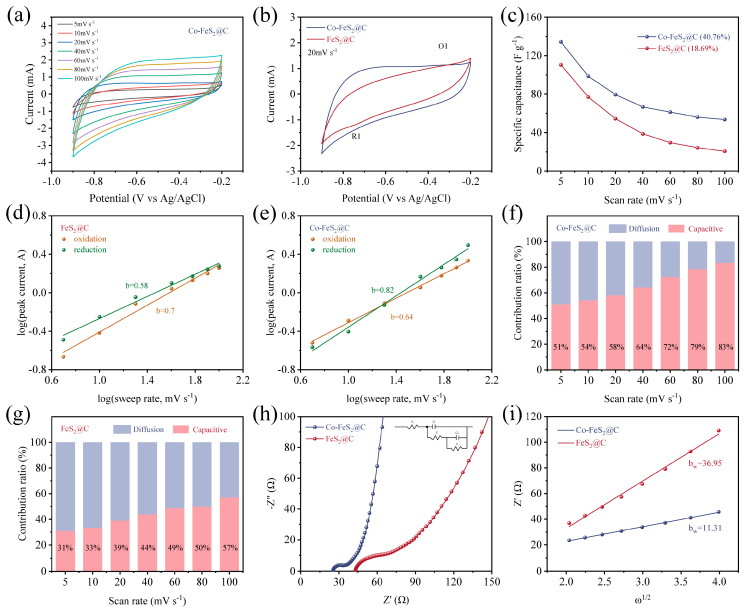
(**a**) CV curves of Co-FeS_2_@C electrode at different scan rates. (**b**) CV curves at a scan rate range of 20 mV s^−1^. (**c**) The specific capacitance at different scan rates for Co-FeS_2_@C and FeS_2_@C. Plot of Log (scan rate) versus Log (peak current) for (**d**) Co-FeS_2_@C and (**e**) FeS_2_@C. Diffusion contribution ratio at different scan rates calculated from CV curves and capacitive for (**f**) Co-FeS_2_@C and (**g**) FeS_2_@C. (**h**) Nyquist plots and (**i**) the corresponding plots of the real part of impedance (Z’) versus the square root of the angular frequency (ω^1/2^) in the Warburg region.

The electrochemical performances of active materials in Yb^3+^ solutions were systematically investigated. As demonstrated in Figure 3a and Figure S6a, CV curves of both two electrodes within a voltage window of −0.9 to −0.2 V (scan rates of 5~100 mV s^−1^) exhibit the quasi-rectangular shapes. Notably, Co-FeS_2_@C retains its rectangular profile even at 100 mV s^−1^, indicating the favorable reaction kinetics and rapid ion diffusion. As displayed in Figure 3b, both electrodes possess distinct redox peaks at 20 mV s^−1^, signifying their pseudocapacitive behavior. The larger CV curve area of Co-FeS_2_@C suggests an increased specific surface area after Co doping. The rate capability analysis reveals that Co-FeS_2_@C retains 40.76% of its capacitance at 5 mV s^−1^, significantly outperforming FeS_2_@C (18.69%), consistent with its enhanced CV stability (Figure 3c). As shown in Figure S6b,c, GCD curves at varying current densities display the symmetrical triangular profiles, confirming high Coulombic efficiency and reversible charge storage behavior. Co-FeS_2_@C demonstrates the prolonged charge–discharge durations and higher specific capacitance compared to FeS_2_@C. As illustrated in Figure S6d, GCD curves at 0.5 A g^−1^ further validate the superior specific capacitance of Co-FeS_2_@C, due to the larger curve areas and the correlated longer charge–discharge times.

To delve deeper into the capacitive electrosorption processes of these two materials, the relationship between peak current (i) and scan rate (v) is analyzed through the following equation [35]:

(2) $$i=av^{b}$$

where b is the slope of the fitted line (Log(i) vs. Log(v)). Typically, a b-value approaching 0.5 or 1 indicates a charge storage mechanism dominated by diffusion-controlled or surface-controlled processes, respectively. As shown in Figure 3d, Co-FeS_2_@C electrode exhibits b-values of 0.82 and 0.64 for its reduction and oxidation peaks, respectively, while the corresponding values for FeS_2_@C are 0.58 and 0.70 (Figure 3e). These results demonstrate that cobalt doping facilitates a transition toward surface-controlled processes (pseudocapacitive behavior) in Co-FeS_2_@C. This shift enhances the electrode’s ability to overcome the limitations associated with diffusion-controlled kinetics, thereby endowing Co-FeS_2_@C with the rapid capacitive storage capabilities.

To further elucidate the influence of Co doping on electrochemical behaviors, the relative contributions of surface-controlled and diffusion-controlled processes are quantified using the equation [58]

(3) $$i=k_{1}v+k_{2}v^{1/2}$$

where $k_{1}$ and $k_{2}$ are adjustable parameters and $k_{1}v$ and $k_{2}v^{1/2}$ present non-diffusion-limited processes and diffusion-limited processes, respectively. As illustrated in Figure 3f,g, the capacitive contribution of Co-FeS_2_@C reaches 51% of total capacitance at a scan rate of 5 mV s^−1^, which is significantly higher than that of FeS_2_@C (31%), suggesting enhanced rapid ion reaction kinetics toward Yb^3+^ after Co doping. Furthermore, this contribution increases to 83% at elevated scan rates. The observed enhancement is primarily attributed to the limited ion diffusion within the electrolyte at higher scan rates, which restricts ionic migration to the electrode surface and thereby promotes a greater proportion of surface-controlled capacitive processes [59,60].

Furthermore, the EIS results of Co-FeS_2_@C in Figure 3h show a smaller semicircle radius and steeper Warburg slope than FeS_2_@C, confirming a lower internal resistance and reduced ion diffusion resistance. The ion diffusion coefficients are calculated based on Nyquist plots. To elucidate the ion diffusion processes within the materials, the ion diffusion coefficients were determined from the Nyquist plots according to the following equation [35,61]:

(4) $$b_{w}=\frac{R^{2}T^{2}}{2A^{2}n^{4}F^{4}C^{2}\sigma^{2}}$$

where R is the gas constant, T is the absolute temperature, A is the surface area of the positive electrode, n is the number of electrons transferred per molecule during oxidation, F is the Faraday constant (96,486 C mol^−1^), C is the concentration, and σ is the Warburg coefficient. The Warburg coefficient σ is related to the diffusion process by the following equation:

(5) $$Z^{\prime }=R_{e}+R_{ct}+\sigma \omega^{1/2}$$

where $R_{e}$ is the interfacial resistance between the electrolyte and the electrode, $R_{ct}$ is the charge transfer resistance, and ω is the angular frequency.

As shown in Figure 3i, Co-FeS_2_@C exhibits a lower Warburg coefficient (b_w_ = 11.31), further confirming its enhanced ion diffusion kinetics. Therefore, the regulation of the lattice structure and electronic configuration of FeS_2_ through cobalt doping significantly improves the transfer rate of Yb^3+^.

### 3.3. CDI of Rare Earth Elements

**Figure 4:**
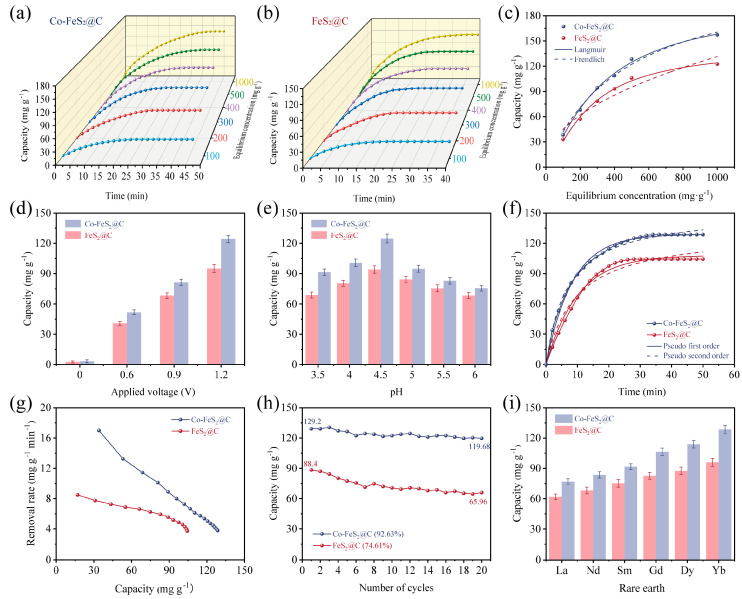
Electrosorption curves at different times and initial concentrations for (**a**) Co-FeS_2_@C and (**b**) FeS_2_@C electrodes. (**c**) Electrosorption isotherms and their nonlinear fitting curves. Electrosorption curves at (**d**) different applied voltages and (**e**) different pH values. (**f**) Kinetics curves and their nonlinear fitting curves. (**g**) Ragon plots. (**h**) Electrosorption–desorption cycling test. (**i**) Electrosorption of different rare earths for Co-FeS_2_@C and FeS_2_@C.

The CDI performances of rare earth elements were systematically investigated using Co-FeS_2_@C and FeS_2_@C, respectively. To evaluate the effect of different initial Yb^3+^ solution concentrations on CDI behavior, various electrosorption experiments were conducted with Yb^3+^ solutions at varying concentrations. As depicted in Figure 4a,b, the electrosorption capacities increase significantly when the initial solution concentrations are elevated. This trend is attributed to enhanced ion transport kinetics and reduced electrical double-layer overlap effects at elevated ion concentrations, leading to improved capacitive performance. Notably, when the initial concentration increases from 500 to 1000 mg L^−1^, both materials exhibit only marginal improvements in their electrosorption capacities, indicating the near-saturation occupancy of active sites at 500 mg L^−1^. In addition, the electrosorption processes were fitted with Langmuir and Freundlich isotherm models, respectively (Figure 4c). The correlation coefficients in Table S1 for the Langmuir model are closer to 1 for these two materials, confirming the dominating monolayer electrosorption mechanism. This suggests that active sites for Yb^3+^ CDI are primarily distributed on the material’s surface. Owing to its superior pore size distribution and enhanced surface diffusion efficiency, Co-FeS_2_@C demonstrates exceptional CDI capacity for Yb^3+^ [62,63].

Furthermore, CDI performance is significantly influenced by the applied voltage. To avoid water splitting (potential of 1.24 V), different voltages are selected to be studied including 0 V, 0.6 V, 0.9 V, and 1.2 V [64]. As shown in Figure 4d, Co-FeS_2_@C exhibits pronounced voltage dependence, with electrosorption capacity increasing markedly at higher voltages. This enhancement is attributed to strengthened electrostatic forces and thickened EDL, improving CDI efficiency. Additionally, elevated voltages promoted redox reactions in FeS_2_, amplifying pseudocapacitive effects and further boosting electrochemical performance.

pH-dependent electrosorption behaviors were evaluated under an initial Yb^3+^ concentration of 500 mg L^−1^ and voltage of 1.2 V (Figure 4e). Electrosorption capacities initially increased when pH values rose from 3.5 to 4.5, because the high H^+^ concentrations induced the competitive occupation of active sites between H^+^ and Yb^3+^ at lower pH. Electrosorption capacity dramatically decreased when the pH value was elevated from 4.5 to 6, mainly due to the reduced H^+^ activity and the diminished material reactivity at higher pH [64].

The kinetics curves of these electrodes reveal that Co-FeS_2_@C achieves an equilibrium electrosorption capacity of 129.2 mg g^−1^, surpassing FeS_2_@C (88.4 mg g^−1^) under an initial Yb^3+^ concentration of 500 mg L^−1^, voltage of 1.2 V, and pH = 4.5 (Figure 4f). Additionally, the fitting analysis in Table S2 reveals that the pseudo-first-order model (higher R^2^) dominates, indicating that the physical electrosorption is governed by electrostatic interactions [65]. The Ragon plot (Figure 4g) highlights the superior electrosorption rate (17.2 mg g^−1^ min^−1^) of Co-FeS_2_@C. As illustrated in Figure 4h, recycling stability tests demonstrate the capacity retention of 92.63% for Co-FeS_2_@C after 20 cycles, outperforming FeS_2_@C (74.61%), which is attributed to Co-induced charge redistribution and enhanced recycling stability. In addition, Figure 4i illustrates the CDI performances of both materials for rare earth ions. Obviously, the electrosorption capacity significantly increases from light rare earth (La^3+^) to heavy rare earth (Yb^3+^), correlating with the decrease in hydrated ionic radii (La^3+^: 1.032 Å → Yb^3+^: 0.868 Å) and increased charge density [4]. The high selectivity of electrode materials for Yb^3+^ is critical for practical applications. To adapt the real application areas, competitive ions (Na^+^ and Ca^2+^) are introduced into a mixed solution containing Yb^3+^, Na^+^, and Ca^2+^, each with an initial concentration of 500 mg L^−1^. As shown in Table S3, the selective electrosorption results reveal only a minor decline in Yb^3+^ electrosorption capacity for both materials under the competitive conditions outlined in Figure S7. This reduction stems from Na^+^ and Ca^2+^ competing for limited active sites and pores on the material surface. Notably, Co-FeS_2_@C exhibits the higher affinity for Yb^3+^, with selectivity following the order of Yb^3+^ > Ca^2+^ > Na^+^. This result is probably due to the stronger electrostatic interactions and coordination between Yb^3+^ and sulfur vacancies on the electrode surface. As shown in Table S4, comparison with previously reported electrode materials reveals the superior electrosorption capacity, durability, and selectivity of Co-FeS_2_@C towards rare earth ions.

### 3.4. CDI Mechanisms

**Figure 5:**
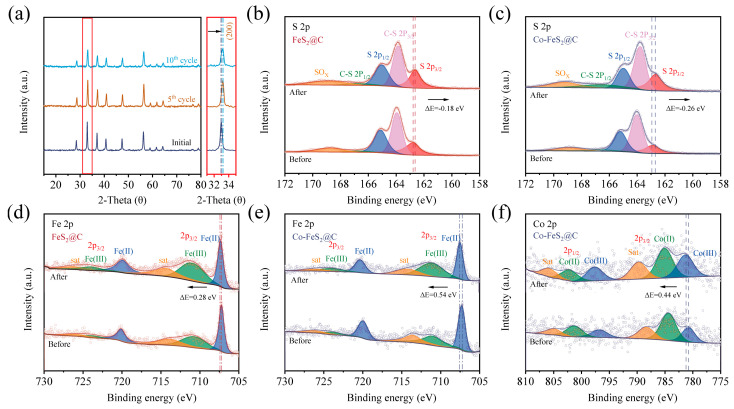
(**a**) XRD patterns of Co-FeS_2_@C electrode after several cycles, XPS spectra of high-resolution spectra of (**b**,**c**) S 2p and (**d**,**e**) Fe 2p of Co-FeS_2_@C and FeS_2_@C electrodes. (**f**) Co 2p of Co-FeS_2_@C electrode.

In order to analyze the CDI mechanism, a variety of characterizations have been performed on the electrode materials after electrosorption tests. As shown in Figure 5a, XRD patterns of Co-FeS_2_@C after the 5th and 10th cycles reveal no characteristic peak changes, confirming that no new species are formed during Yb^3+^ electrosorption [27]. However, the magnified XRD patterns exhibit a positive shift in characteristic peaks for post-electrosorption, indicating lattice contraction. Due to the larger initial lattice spacing enabled by Co doping, Co-FeS_2_@C effectively mitigated structural strain during electrosorption–desorption cycles, enhancing cycling stability. As shown in Figure S8a, XPS analysis of Yb^3+^-loaded electrode materials shows a new F 1s peak at 690.2 eV, which is attributed to PVDF binder that is used in the preparation of the working electrode. As discerned from Figure S8b, the distinct Yb 4d_5/2_ peaks at 186.5 eV and Yb 4d_3/2_ at 193.2 eV confirm the successful electrosorption of Yb^3+^. Additionally, another characteristic peak corresponding to the Yb-S bond is observed, further evidencing the chemisorption process between the material and Yb^3+^ ions [64]. In addition, the high-resolution C 1s spectra (Figure S8c,d) reveal the emergence of an additional C=O bond after electrosorption, which is attributed to partial oxidation during the electrode drying process. Furthermore, the comparative analysis of Fe 2p and S 2p spectra (Figure 5b–e) demonstrates peak shifts after electrosorption process. The S 2p peak shifts toward the lower binding energy, suggesting increased electron density at S sites due to electron gain during electrosorption. Conversely, the Fe 2p peaks shifts to a higher binding energy, reflecting reduced electron density as Fe^2+^ is oxidized to Fe^3+^. Quantitative peak area analysis confirms a decrease in Fe^2+^ content and an increase in Fe^3+^, indicating chemical state evolution. After Co, with its lower electronegativity, is doped into FeS_2_, the charge-driving forces are amplified during the electrosorption process, accelerating the reaction kinetics. As shown in Figure 5f, the Co 2p spectra further reveal that Co^2+^ is also oxidized to Co^3+^, synergistically enhancing electron transfer and redox activity.

**Figure 6:**
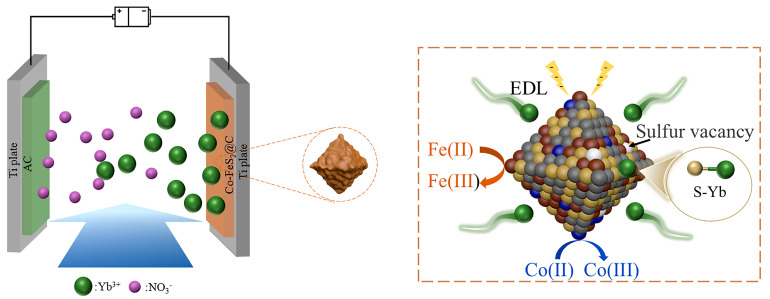
Schematic diagram of CDI process of Yb^3+^ for Co-FeS_2_@C.

As illustrated in Figure 6, the electrosorption processes in both two materials are predominantly attributed to the synergistic physico-chemical electrosorption mechanism: (i) the electric double-layer (EDL) effect at the material interface; (ii) pseudocapacitive contributions from FeS_2_ redox reactions; and (iii) chemical coupling between surface sulfur atoms and Yb^3+^ ions via S-Yb bond formation, collectively significantly improving electrosorption capacity and selectivity. In addition, for Co-FeS_2_@C, Co doping further augments electrosorption performance through a dual-functional mechanism: (1) modulating electron density around Fe sites to strengthen electrochemical driving forces and accelerate mass transfer and (2) introducing sulfur vacancies in FeS_2_ to enhance redox activity. This synergistic dual-effect mechanism cooperatively optimizes both electrosorption kinetics and capacity, establishing Co-FeS_2_@C as an exceptionally promising candidate for high-efficiency rare earth ion recovery electrodes.

## 4. Conclusions

In conclusion, cobalt dopants were successfully incorporated into an MIL-101(Fe) framework through a one-step hydrothermal synthesis. Subsequently, a MOF-derived carbon–sulfide composite, termed Co-FeS_2_@C, was fabricated via the carbonization–sulfidation process. Comparative analysis revealed that cobalt doping effectively expanded the lattice spacing and introduced additional defects in FeS_2_@C, facilitating the exposure of more active sites and optimizing electron transfer kinetics. Electrochemical characterizations confirmed that Co doping increased the surface-controlled contribution from 57.1% to 83.3%, enhancing EDL behavior and consequently improving mass transport efficiency. Under the optimized CDI conditions, the Co-FeS_2_@C electrode exhibited a superior eletrosorption capacity of 129.2 mg g^−1^ and with an improved CDI capacity retention of 92.63% after 20 cycles. In addition, kinetics and isotherm analysis demonstrated that both electrodes followed a monolayer physico-chemical synergistic electrosorption mechanism. XPS analysis before and after electrosorption revealed that the enhanced CDI performance of Co-FeS_2_@C primarily originated from the higher electronegativity of the incorporated cobalt, and the optimized electronic structural modulation. Therefore, the electron density around iron sites is reduced, providing a stronger charge-driving force during CDI, ultimately leading to a simultaneous improvement in both electrosorption kinetics and capacity. This work provides a novel strategy and deep insights for enhancing electrosorption performances.

## Data Availability

The original contributions presented in this study are included in the article/Supplementary Material. Further inquiries can be directed to the corresponding authors.

## Supplementary Materials

The following supporting information can be downloaded at https://www.mdpi.com/article/10.3390/ma18214994/s1, Figure S1: XRD patterns of (a) Co-MIL-101 and MIL-101, (b) Co-Fe_3_O_4_ and Fe_3_O_4_; Figure S2: SEM images of (a,b) MIL-101(Fe) and (c,d) MIL-101(Co, Fe); Figure S3: FTIR spectroscopy of MIL-101(Fe) and MIL-101(Co, Fe). (In the spectrum, the characteristic peaks at 544, 1388, 1600, and 1660 cm^−1^ correspond to the stretching vibrations of metal-oxygen bonds (Fe-O/Co-O), the symmetric vibration of C-O bonds, the asymmetric vibration of C=O bonds, and the stretching vibrations of C=O bonds, respectively); Figure S4: (a) SEM images and (b) TEM images of FeS_2_@C; Figure S5: Co 2p spectra of Co-FeS_2_@C electrode; Figure S6: FeS_2_@C electrode: (a) CV curves at a scan rate range of 5–100 mV s^−1^, (b,c) GCD curves at 0.5, 1 and 2 A g^−1^, (d) GCD curves at 0.5 A g^−1^ of FeS_2_@C and Co-FeS_2_@C electrodes; Figure S7: The electrosorption capacities of two electrodes for Na^+^, Ca^2+^ and Yb^3+^ in the competitive solution. Figure S8: XPS spectra of FeS_2_@C and Co-FeS_2_@C electrodes: (a–c) high-resolution spectra of Yb 4d and C 1s. (d) Co 2p of Co-FeS_2_@C electrode; Table S1: Isotherm parameters using Langmuir and Freundlich models; Table S2: Kinetic parameters for electrosorption of Yb^3+^ for two electrodes; Table S3: Electrosorption capacities and separation factors of two electrodes for Na^+^, Ca^2+^ and Yb^3+^; Table S4: Comparison of studied electrodes and previous materials for electrosorption of metal ions. References [66,67,68,69,70,71,72,73,74,75] are cited in the Supplementary Materials.
