# Supplementary material for: Electronic Structure and Lattice Engineering of Cobalt Doping FeS2@C for Superior Electrosorption of Ytterbium Ions
Source: Materials (Basel). 2025 Oct 31;18(21):4994. doi: 10.3390/ma18214994 (PMC12610281; doi:10.3390/ma18214994)
Supplement: Supplementary file 1 [file materials-18-04994-s001.zip › materials-3928907-supplementary.pdf]

Supplementary Materials

Electronic structure and lattice engineering of cobalt doping FeS_2@C for superior electrosorption of ytterbium ions

Kaicheng Bi [1], Tiancai Cheng [1], Zhangjie Shi [1], Wenyan Huang [1], Fuli Deng [1]*, Yi Zhang [1,2]*

[1] State Key Laboratory of Green and Efficient Development of Phosphorus Resources, Key Laboratory of Green Chemical Process of Ministry of Education, Hubei Key Laboratory for Novel Reactor and Green Chemical Technology, Engineering Research Center of Phosphorus Resources Development and Utilization of Ministry of Education, School of Chemical Engineering and Pharmacy, Wuhan Institute of Technology, Wuhan 430073, China

[2] Hubei Three Gorges Laboratory, Yichang 443007, China

Corresponding Author

Yi Zhang, E-mail: zhangyi_1208@whu.edu.cn; Fuli Deng, E-mail: fldeng@wit.edu.cn.

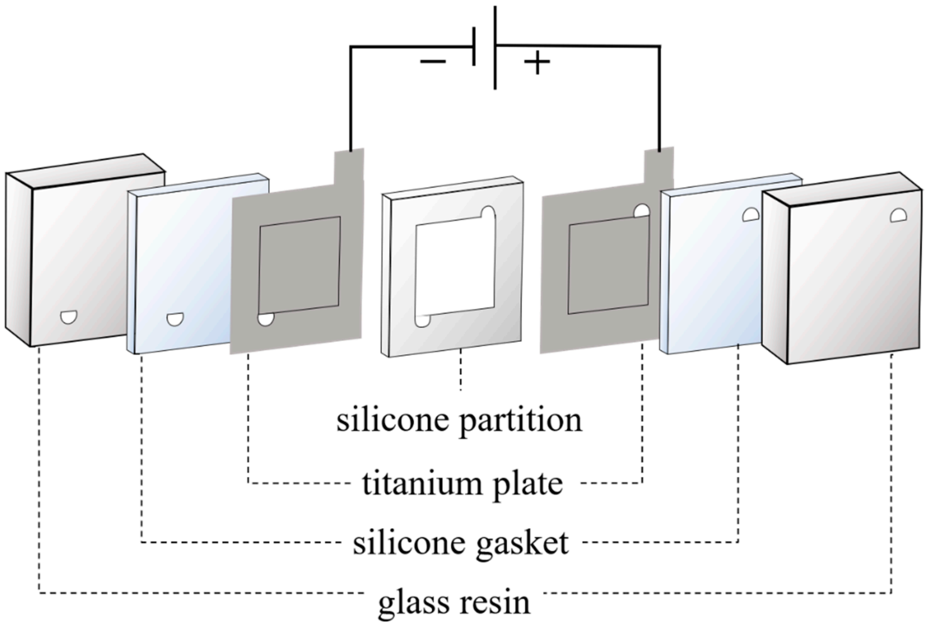

Schematic diagram of CDI device for electrosorption of rare earth ions.

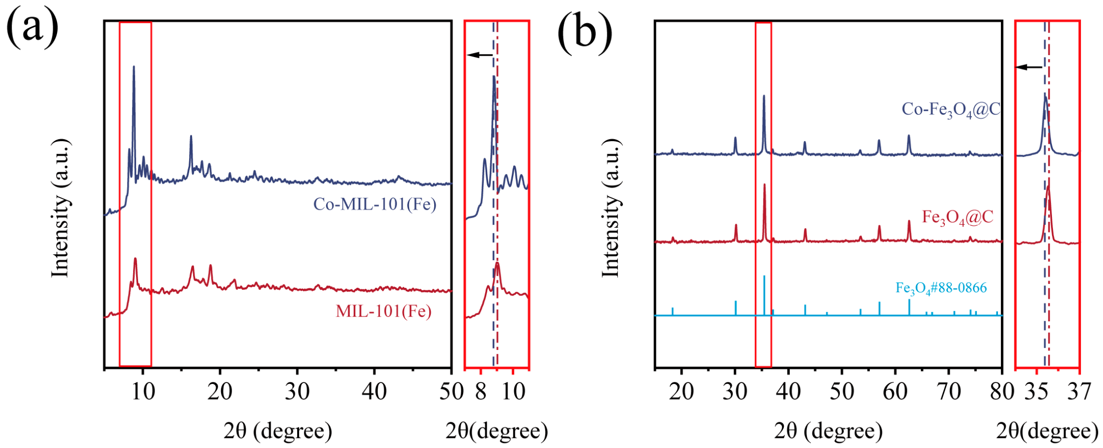

Figure S1. XRD patterns of **(a)** Co-MIL-101 and MIL-101, **(b)** Co-Fe_3O_4 and Fe_3O_4.

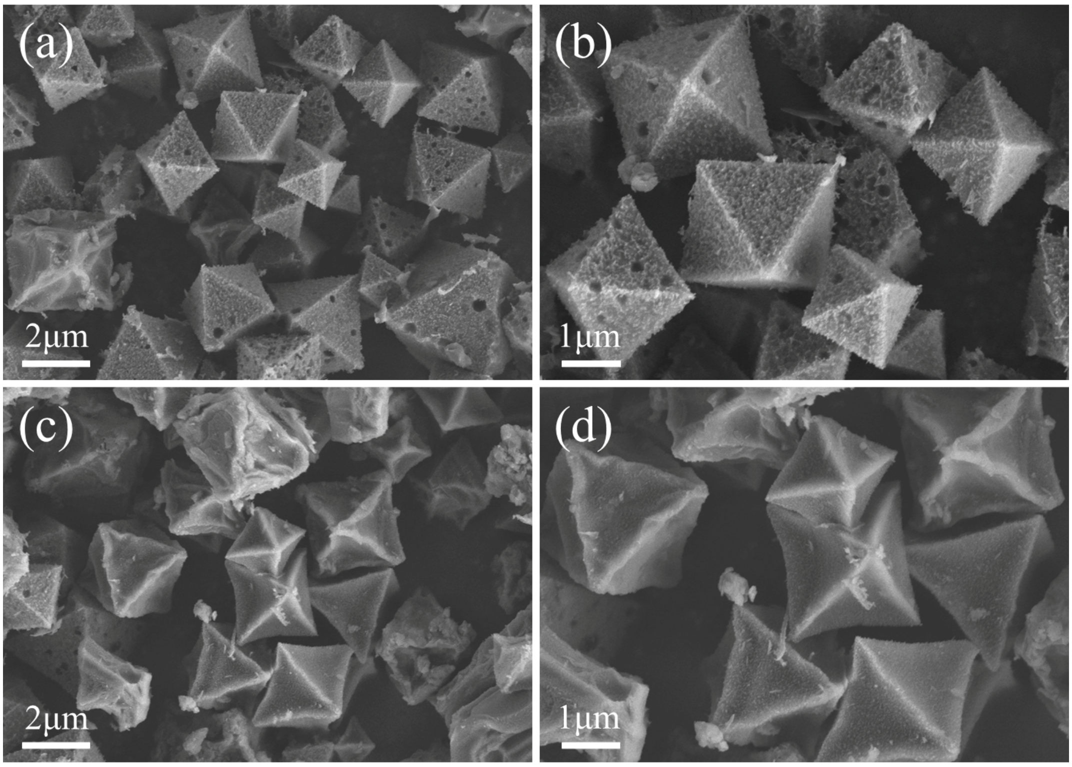

Figure S2. SEM images of **(a, b)** MIL-101(Fe) and **(c, d)** MIL-101(Co, Fe).

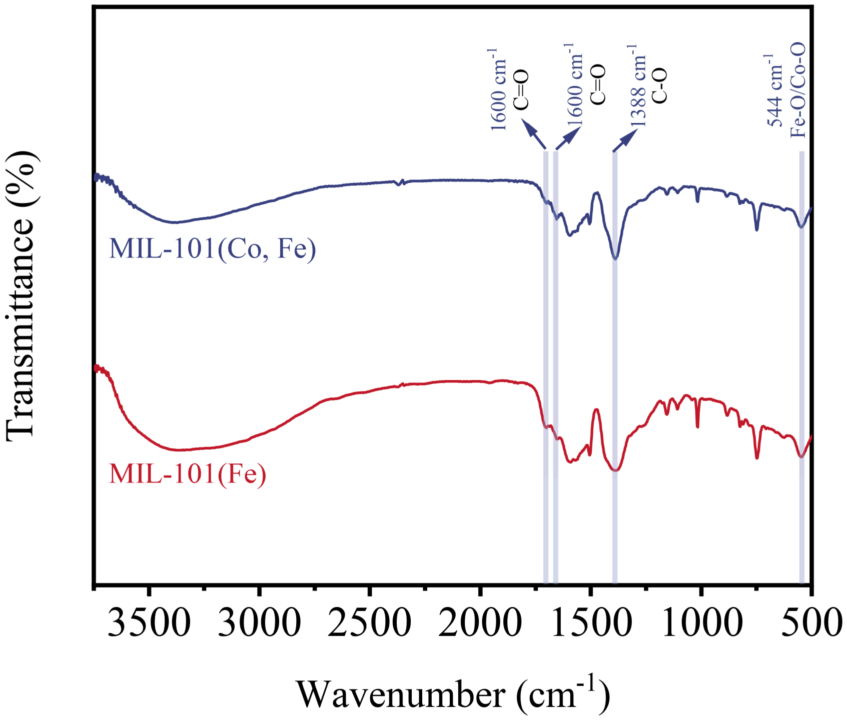

Figure S3. FTIR spectroscopy of MIL-101(Fe) and MIL-101(Co, Fe). (In the spectrum, the characteristic peaks at 544, 1388, 1600, and 1660 cm^{-1} correspond to the stretching vibrations of metal-oxygen bonds (Fe–O/Co–O), the symmetric vibration of C–O bonds, the asymmetric vibration of C=O bonds, and the stretching vibrations of C=O bonds, respectively.)

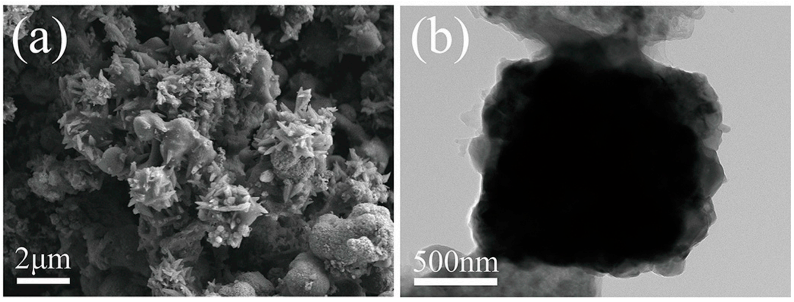

Figure S4. (a) SEM images and **(b)** TEM images of FeS_2@C.

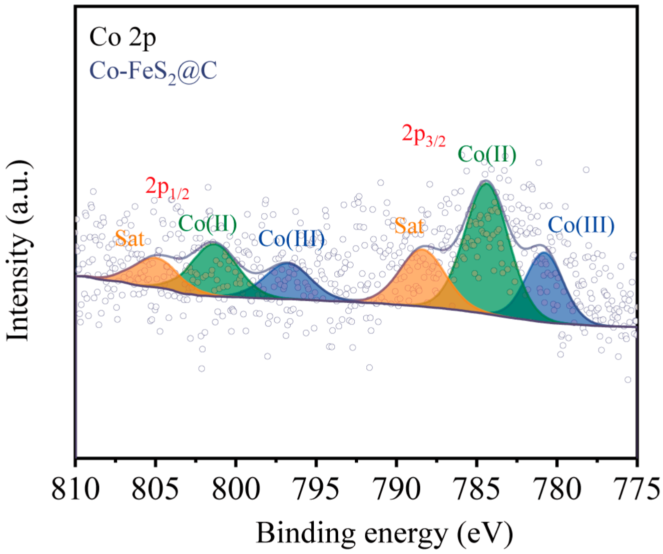

Figure S5. Co 2p spectra of Co-FeS_2@C electrode.

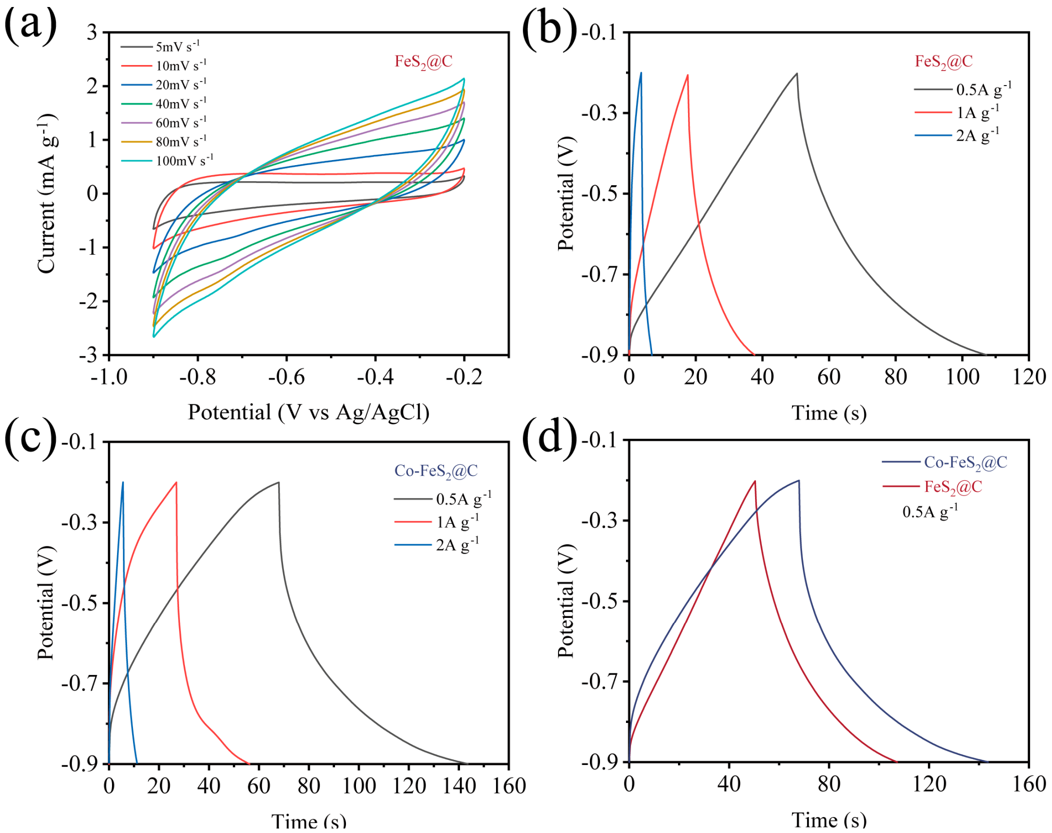

Figure S6. FeS_2@C electrode: **(a)** CV curves at a scan rate range of 5-100mV s^{-1}, **(b, c)** GCD curves at 0.5, 1 and 2 A g^{-1}, **(d)** GCD curves at 0.5 A g^{-1} of FeS_2@C and Co- FeS_2@C electrodes.

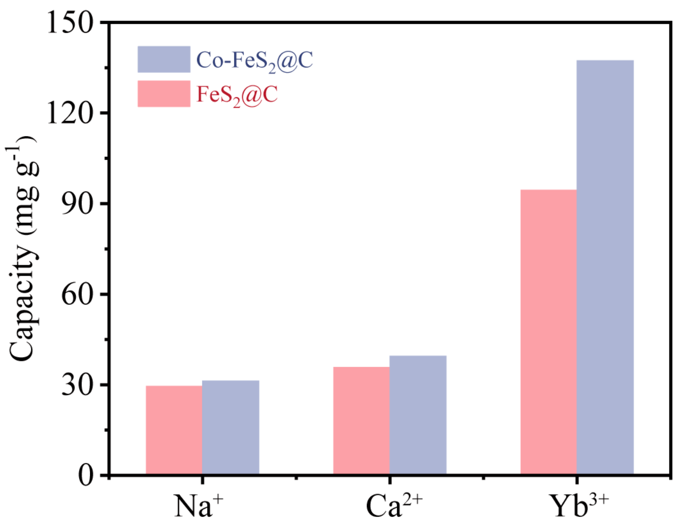

Figure S7. The electrosorption capacities of two electrodes for Na^+, Ca^{2+} and Yb^{3+} in the competitive solution.

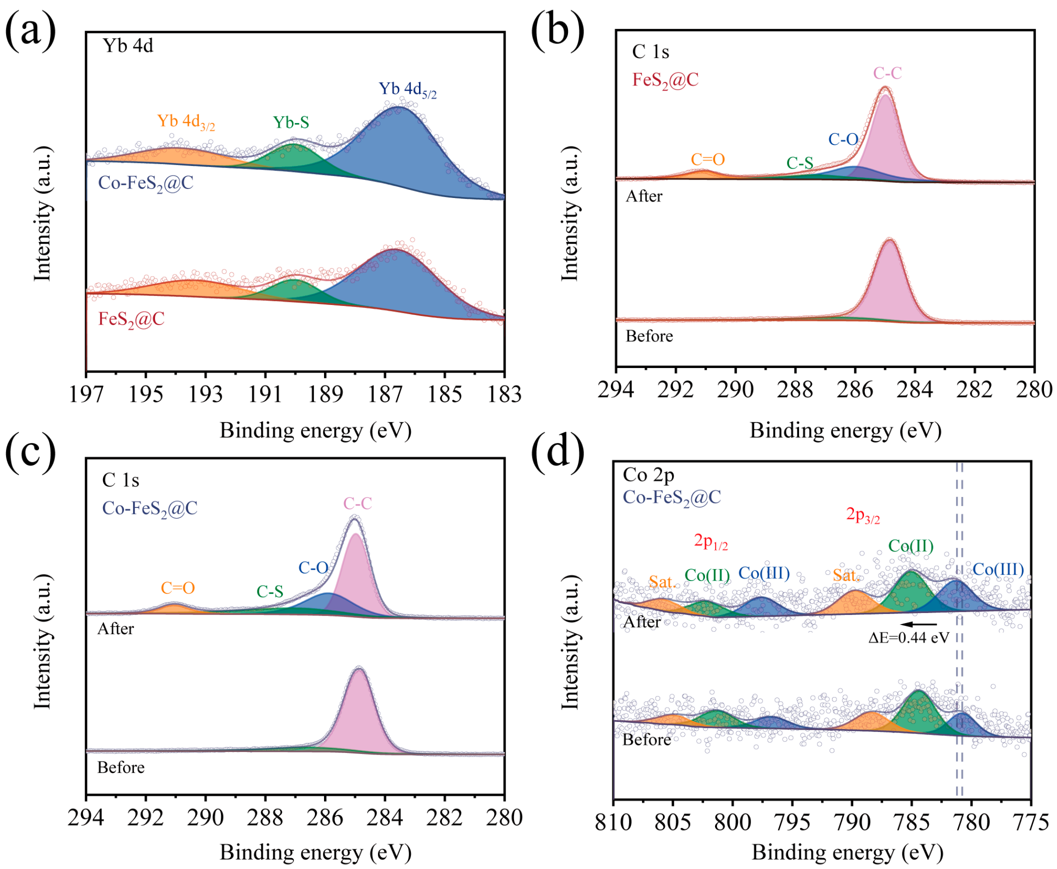

Figure S8. XPS spectra of FeS_2@C and Co-FeS_2@C electrodes: **(a-c)** high-resolution spectra of Yb 4d and C 1s. **(d)** Co 2p of Co-FeS_2@C electrode.

Table S1 Isotherm parameters using Langmuir and Freundlich models.

Samples	Langmuir model		Freundlich model		
	K_L (L mg^{-1})	R^2	K_F (mg g^{-1}) (L mg^{-1})1/n	1/n	R^2
FeS_2@C	4.679×10^{-4}	0.99158	5.262	0.46844	0.87052
Co-FeS_2@C	8.204×10^{-4}	0.99746	5.168	0.49131	0.94676

For isotherm analysis, Langmuir and Freundlich models are applied to fit the isothermal curves and illustrated as follows:

$$q_e = \frac{q_m K_L C_e}{1 + K_L C_e} \qquad \text{S1}$$

$$q_e = K_F C_e^{1/n} \qquad \text{S2}$$

where the equilibrium capacity and maximal capacity are referred to q_e and q_m (mg g^{-1}), C_e represents the equilibrium concentration, respectively. K_L is a parameter of Langmuir isothermal model. K_F and n are the parameters of Freundlich isothermal model.

Table S2 Kinetic parameters for electrosorption of Yb^{3+} for two electrodes.

Samples	Pseudo first order			Pseudosecond order		
	k_1 (min^{-1})	q_e (mg g^{-1})	R^2	k_2 (g mg^{-1}·min^{-1})	q_e (mg g^{-1})	R^2
FeS_2@C	0.1194	108.04	0.9907	0.966×10^{-3}	132.32	0.9689
Co-FeS_2@C	0.1001	128.86	0.9954	0.811×10^{-3}	154.49	0.9953

The CDI kinetics are fitted with pseudo first order and pseudo second order models, respectively, which are demonstrated in the following equations.

$$\ln(q_e - q_t) = \ln q_e - k_1 t \qquad \text{S3}$$

$$\frac{t}{q_t} = \frac{1}{k_2 q_e^2} + \frac{t}{q_e} \qquad \text{S4}$$

where q_e and q_t (mg g^{-1}) represents the CDI capacity at equilibrium and time t. Besides, k_1 (min^{-1}) and k_2 (g mg^{-1} min^{-1}) are the kinetics constants of pseudo first order and pseudo second order, respectively.

Table S3 Electrosorption capacities and separation factors of two electrodes for Na^{+}, Ca^{2+} and Yb^{3+}.

Samples	Na^{+} (mg g^{-1})	Ca^{2+} (mg g^{-1})	Yb^{3+} (mg g^{-1})	Yb^{3+}/ Na^{+}	Yb^{3+}/ Ca^{2+}
FeS_2@C	29.463	35.781	94.485	3.00	2.29
Co-FeS_2@C	31.283	39.468	137.358	4.10	3.02

The selective electrosorption behavior of the three electrodes in the CDI cell is characterized using the ion selectivity coefficient, whose governing equation is presented below:

$$\alpha_{\mathrm{M}}^{Yb^{3+}}=\left(\frac{C_{Yb^{3+},\mathrm{initial}}-C_{Yb^{3+},\mathrm{final}}}{C_{\mathrm{M,initial}}-C_{\mathrm{M,final}}}\right)\left(\frac{C_{\mathrm{M,initial}}}{C_{Yb^{3+},\mathrm{intial}}}\right) \qquad \text{S5}$$

Where M represents the competition metal ions Na^{+} and Ca^{2+}, while $C_{M,initial}$ and $C_{M,final}$ (mg g^{-1}) are denoted as the initial and final concentrations of ions in the multi-salt solution. Similarly, $C_{Yb^{3+},initial}$ and $C_{Yb^{3+},final}$ (mg g^{-1}) refer to the initial and final concentrations of Yb(III) in the multi-salt solution ($C_{Yb^{3+},\mathrm{intial}}$ =518 mg g^{-1}; $C_{Ca^{2+},\mathrm{initial}}$=453mg g^{-1} and $C_{Na^{+},\mathrm{initial}}$=487 mg g^{-1}).

Table S4. Comparison of studied electrodes and previous materials for electrosorption of metal ions.

Materials	Metal	Capacity $mg\cdot g^{-1}$	Retention/cycles	selectivity	Reference
PCNFs-SZ	Na^+	37.09	75.0%/30	/	[1]
FeNC	Na^+	24.95	86.39%/10	/	[2]
MIIM	Fe^{3+}	45.4	79.0%/7	/	[3]
Fe_3O_4NPs/HPC	Cd^{2+}	34.22	100%/5	1.18(Cd^{2+}/Na^+	[4]
PC-900	La^{3+}	88.89	71.7%/10	1.91(La^{3+}/Na^+)	[5]
PPyN-AC	La^{3+}	23.6	100%/5	2.33(La^{3+}/Na^+)	[6]
MnO@PC-900	La^{3+}	93.02	54.12%/15	1.98(La^{3+}/Na^+)	[7]
Carbon	Nd^{3+}	125	94%/6	1.1(Nd^{3+}/Na^+)	[8]
P(FPMAm-co-MAA)	Y^{3+}	69.4	100/4	/	[9]
NiFeP@NC	Yb^{3+}	105.24	90.3%/10	1.78(Yb^{3+}/Na^+)	[10]
FeS_2@C	Yb^{3+}	88.4	78.46%/10	3.00(Yb^{3+}/Na^+)	This work
Co-FeS_2@C	Yb^{3+}	129.2	94.73%/10	4.10(Yb^{3+}/Na^+)	This work

Reference

1. Ma, X.; Wang, J.; Zhu, Z.; Wang, N.; Wang, C.; Nie, G., A two-pronged strategy to boost the capacitive deionization performance of nitrogen-doped porous carbon nanofiber membranes. *Desalination.* **2025**, 594. [CrossRef]
2. Liu, S.; Zhang, P.; Wang, Y.; He, M.; Zhang, W.; Xu, Z.; Li, K., Uniformly Dispersed Fe–N Active Centers on Hierarchical Carbon Electrode for High-Performance Capacitive Deionization: Plentiful Adsorption Sites and Conductive Electron Transfer. *ACS Sustain. Chem. Eng.* **2023**, 11, (24), 8847-8857. [CrossRef]
3. Hu, Q.-h.; Tang, D.-y.; Xiang, Y.-l.; Chen, X.; Lin, J.; Zhou, Q.-h., Magnetic ion-imprinted polyacrylonitrile-chitosan electro-spun nanofibrous membrane as recyclable adsorbent with selective heavy metal removal and antibacterial fouling in water treatment. *Int. J. Biol. Macromol.* **2023**, 241, 124620. [CrossRef]
4. Wang, N.; Wang, M.; Quan, H.; Wang, S.; Chen, D., Waste Camellia oleifera shell-derived hierarchically porous carbon modified by Fe_3O_4 nanoparticles for capacitive removal of heavy metal ions. *Sep. Purif. Technol.* **2024**, 329. [CrossRef]
5. Xiong, Y.; Yang, X.; Liu, Y.; Chen, X.; Wang, G.; Lu, B.; Lin, G.; Huang, B., Fabrication of phosphorus doping porous carbon derived from bagasse for highly-efficient removal of La^{3+} ions via capacitive deionization. *Electrochim. Acta.* **2022,** 404, 139735. [CrossRef]
6. Zhao, F.; Chen, S.; Xiang, H.; Gao, T.; Wang, D.; Wei, D.; Sillanpää, M.; Ke, Y.; Tang, C.-J., Selectively capacitive recovery of rare earth elements from aqueous solution onto Lewis base sites of pyrrolic-N doped activated carbon electrodes. *Carbon.* **2022**, 197, 282-291. [CrossRef]
7. Lin, G.; Wang, G.; Xiong, Y.; Li, S.; Jiang, R.; Lu, B.; Huang, B.; Xie, H., High-performance electrosorption of lanthanum ion by Mn_3O_4-loaded phosphorus-doped porous carbon electrodes via capacitive deionization. *J. Environ. Manage.* **2024**, 358, 120856. [CrossRef]
8. Engmann, E.; Diaz, L. A.; Lister, T. E.; Atifi, A.; Palasyuk, O.; Zhao, H., Selective pseudocapacitive immobilization of REE elements on carbon based electrodes. *Electrochim. Acta.* **2024**, 478. [CrossRef]
9. Vapnik, H.; Elbert, J.; Su, X., Redox-copolymers for the recovery of rare earth elements by electrochemically regenerated ion-exchange. *J. Mater. Chem. A.* **2021**, 9, (35), 20068-20077. [CrossRef]
10. Zhang, Y.; Ji, Y.; Sun, C.; Bi, K.; Wang, H.; Ding, Y., Selective electro-capacitive deionization of Yb(III) by nanospherical N-doped carbon supported nickel-iron bimetallic oxide, nitride and phosphide. *Desalination.* **2024**, 592, 118104. [CrossRef]
